# Identifying the factors affecting financial toxicity status in patients with middle and advanced colorectal cancer: a cross-sectional study

**DOI:** 10.3389/fpubh.2024.1421314

**Published:** 2024-07-16

**Authors:** Xiaofang He, Jie Chen, Lin Zhang, Qiuping Li, Xiaoli Zhu, Jie Zhao, Ying Chen

**Affiliations:** ^1^Medical School, Jiangnan University, Wuxi, China; ^2^Department of Nursing, Guizhou Provincial People's Hospital, Guiyang, China; ^3^The Second Affiliated Hospital of Guizhou University of Chinese Medicine, Guiyang, China; ^4^Department of Oncology, Affiliated Hospital of Jiangnan University, Wuxi, China

**Keywords:** middle and advanced CRC, determinants, Influencing factors, financial toxicity, survey

## Abstract

**Background:**

Colorectal cancer (CRC) ranks as the second most prevalent type of cancer in China. The financial implications of treatment are a significant factor to be taken into account for patients diagnosed with middle and advanced stages of colorectal cancer (III-IV CRC). The research aims to explore current financial toxicity (FT) conditions and analyze factors that may influence it in patients with middle and advanced CRC.

**Method:**

This is a cross-sectional survey. The participants of the study were individuals diagnosed with middle and advanced colorectal cancer who were admitted to the hospital between January and June 2023. The cross-sectional survey utilized a variety of instruments, including a general information questionnaire, a cancer patient report outcome economic toxicity scale, a medical coping style questionnaire, an Anderson symptom assessment scale, a disease shame scale, and a social support scale. Multiple linear regression analysis was employed to examine the factors influencing FT.

**Result:**

A cohort of 264 patients diagnosed with stage III-IV CRC were included in the study. The majority of patients with intermediate and advanced CRC (87.1%, *n* = 230) reported experiencing substantial financial strain. Multivariate analysis revealed that factors influencing FT included low family monthly income, out-of-pocket expenses, unemployment, undergoing surgical treatment, the level of stigma, and the severity of symptoms (*P* < 0.001).

**Conclusion:**

Patients with stage III-IV cancer (CRC) demonstrate increased levels of financial toxicity (FT), a common occurrence in individuals with moderate to severe CRC. In patients with stage III-IV CRC, the presence of FT is correlated with various factors including family monthly income, medical payment methods, work status, surgical treatment, stigma levels, and symptom severity. These characteristics may serve as influencing factors for subsequent treatment decisions.

## Introduction

Based on the global cancer burden data, it was reported that in 2020 there were an estimated 1.93 million new cases and 940,000 deaths attributed to CRC (1). CRC ranks as the third most prevalent cancer worldwide and the second highest cause of cancer-related mortality. In China, CRC is the second most prevalent cancer, with projections indicating a significant increase in cases from 0.56 million in 2020 to 0.91 million in 2040, representing a 64% rise (2, 3). Unfortunately, colorectal cancer often goes undetected in its early stages, resulting in most patients being diagnosed at advanced stages (4). Significant advancements have been made in the understanding and treatment of this disease over the last few decades. These include targeted therapy, immunotherapy, chemoradiotherapy, endoscopic and surgical excision, and multimodal interventions (3, 5). Multiple therapy modalities work together to dramatically slow tumor development and increase survival time for individuals with middle and advanced CRC.

Nevertheless, One study reported the cost of CRC management $2,045–10,772 per year in direct medical costs and $551–795 per year in indirect medical costs (6). The cost of treating CRC places a significant financial burden both individuals and healthcare systems. A term associated with this situation is “financial toxicity” (7). The Financial toxicity, also known as FT, refers to substantial costs due associated with cancer treatment. FT is not only the objective burden on treatment costs, but also includes the economic pressure and living difficulties experienced by patients and their families (8). The expenditures of therapy, examination, accommodations, commuting, and indirect lost time due to labor costs are examples of objective financial burdens (9). The financial distress increase in treatment costs and financial difficulties for families can lead to anxiety, depression, and other emotions for patients and their families. It has been shown that FT not only leads to medical delays or cancellations, but also leads to a decrease in patient adherence to treatment, which in turn negatively affects disease prognosis and quality of life (10–13). Thus, FT has emerged as a challenging obstacle to public health initiatives across the world.

FT has emerged as a serious financial problem in the world and is receiving increasing attention (14). In addition to the type of disease, many factors affect the level of FT. Previous research has shown that the incidence of FT associated with cancer might vary from 28 to 80%, 80% had material burden and 34.3% reported psychological challenges (10, 15, 16). According to the research, patients from low-income households are more likely to endure FT, and are more likely to be unemployed, and have less savings to compensate for the cost of treatment (17). Additionally, FT may be influenced by a range of treatment-related and clinicopathologic factors. Among breast cancer patients, FT was associated with employment status, insurance status, baseline financial status, tumor stage, and type of surgery (18–21). Nevertheless, FT's impact on individuals with middle and advanced CRC is not yet known.

Although the majority of research have focused on breast cancer, lung cancer, and prostate cancer (22–24); there is a scarcity of research of studies on the role of FT in middle and advanced CRC. Furthermore, the specific psychological burden experienced by patients with middle and advanced CRC requires further consideration. Consequently, the present Chinese- based study sought to: evaluate the present status of FT in patients with CRC in its middle to late stages, elucidate the underlying factors, and improve the coping abilities of patients with CRC.

## Methods

### Design and sample

This is a cross-sectional study. The survey was conducted in a tertiary hospital in Guizhou Province, over the course of January to June 2023. According to the pre survey and the sample size formula, the sample size is estimated using the given equation (25): $n=\frac{\left(Z_{\alpha /2}\right)2}{\left(\delta \right)2}\cdot \pi \left(1-\pi \right)$, where *n r*epresents the initial sample size estimation and *Z* represents the confidence level (α), The prevalence is illustrated and is a marginal error. The prediction test data showed that the FT rate was as high as 87%. The minimum sample size was calculated by taking α = 0.01 and δ = 0.05, resulting in a total sample size of 174 participants. The effect of an overall sampling design of 1.5 and a sample size of at least 261 participants was taken into account. Considering a sample failure rate of 5–10%, 280 patients are planned to be recruited. The inclusion criteria for this study were as follows: (1) patients had to be at least 18 years old at the time of diagnosis; (2) patients had to be staged with middle and advanced (III-IV) CRC; (3) patients had to demonstrate sufficient reading and writing abilities; (4) patients had to be capable of completing the survey independently or with guidance. (5) Patients provided informed consent and signed consent forms. Individuals with a prior mental health condition or history of multiple cancer diagnoses were excluded from participation. Approval for this study was obtained from the hospital's Ethics Committee [Ethics Review No. Lun Audit (Research) 2023-036].

### Data collection and measurements

Six questionnaires were utilized in the study. A 17-item questionnaire was employed to collect sociodemographic and clinicopathological data. The FT was assessed using the 11-item Comprehensive Score for Financial Toxicity (COST) questionnaire (26). The Chinese version of the COST-PROM demonstrated a high level of reliability, with a Cronbach's α coefficient of 0.889. Financial Toxicity was categorized into high FT (score < 22) and low financial toxicity FT (score ≥ 22) by B. Zeybek and Huihui Yu from Yale University, with lower scores indicating higher levels of financial toxicity (27, 28). Additionally, the Medical Coping Modes Questionnaire was also administered. The Medical Coping Modes Questionnaire (MCMQ) scale was employed, comprising a total of 20 items across the three domains of submission, avoidance, and confrontation (29). The three aspects of the questionnaire have Cronbach's α values of 0.76, 0.60, and 0.69, respectively. The M.D. contains a total of 19 items. Anderson Symptom Inventory (MDASI) (30) scale, which is divided into two sections: degree of distress and symptom severity with respective Cronbach's α coefficients of 0.959 and 0.970. The Stigma Scale for Chronic Illness (SSCI) evaluates the level of stigma experienced by individuals with chronic illnesses using 24 items that measure both intrinsic and extrinsic stigma (31). The Cronbach's α coefficient for the scale was calculated to be 0.951, indicating high internal consistency. Additionally, the Social Support Rating Scale (SSRS) (32) was utilized, comprising 10 items that assess subjective support, objective support, and social support utilization. The scale demonstrated strong validity and reliability, as evidenced by a Cronbach's α value of 0.92.

### Statistical analysis

The data underwent sorting, counting, and analysis utilizing SPSS version 26.0 for statistical purposes. Enumeration data were presented as [*n* (%)]. The measurement data that obeyed the normal distribution was described by $\bar{x}\pm s$. Normality of measurement data was initially assessed through the Shapiro-Wilk test. Analysis of variance was employed to compare and examine differences in normal indicators across multiple groups, with *post-hoc* pairwise comparisons conducted utilizing the S-N-K method for intergroup distinctions. *Post-hoc* pairwise comparisons were conducted utilizing the Student-Newman-Keuls (S-N-K) method to assess intergroup variances. Multiple linear regression was employed to examine the factors influencing the scores on the FT scale. The significance level for all analyses was set at α = 0.05, with statistical significance defined as *P* < 0.05.

## Results

### Clinical and demographic variable distribution

We recruited a total of 280 cases, 264 of which ultimately participated, with a participation rate of 94%. As shown in Table 1, the baseline characteristics of the entire cohort and the description of its cost score can be displayed. The average age of the study subjects was 56.70 ± 14.70 years, of which 152 (57.6%) were male. Two hundred and thirty cases (87.1%) had high ft, and 216 cases (81.8%) were Han. One hundred and fourteen (43.2%) families had a monthly income between 2,000 and 5,000 yuan, and 217 (82.2%) were married. Ninety-three cases (35.2%) were in junior high school. One hundred and sixty-three (61.7%) patients preferred medical insurance payment. Ninety-seven cases (36.7%) were retired. Two hundred and thirty-seven cases (89.8%) underwent surgical treatment as the main treatment. The majority were only children in 100 cases (37.9%). One hundred and seventy-one cases (64.8%) had a disease duration of ≤ 1 year. More than half of 133 cases (50.4%) were hospitalized ≤ 2 times. Eighty-four cases (31.8%) had chronic diseases. One hundred and fifty-one cases (57.2%) took more than 1 h to go to the medical center by car. More than half of 136 cases (51.5%) spent more than 100,000 US dollars on medical expenses, and 147 cases (59.8%) were in clinical stage III.

**Table 1:** Basic characteristics of patients with middle- to late-stage CRC (*n* = 264).

**Norm**	**Categorization**	**Precedent [*n* (%), $\bar{x}\pm s$]**
FT classification	High FT	230 (87.1)
	Low FT	34 (12.9)
Sex	Male	152 (57.6)
	Female	112 (42.4)
Race	Han ethnic group	216 (81.8)
	Other ethnic groups	48 (18.2)
Monthly household income (yuan/month)	< 2,000	71 (26.9)
	2,000–5000	114 (43.2)
	>5,000	79 (29.9)
Marital status	Married	217 (82.2)
	Unmarried/divorced/widowed	47 (17.8)
Education level	Primary and below	57 (21.6)
	Junior high school	93 (35.2)
	High school/secondary school	68 (251.8)
	University and above	46 (17.4)
Payment methods	Self-funded or other	13 (4.9)
	Medical insurance	163 (61.7)
	New Agriculture Cooperative Program (NAC)	88 (33.3)
Employment status	Employed	35 (13.3)
	Unemployed	40 (15.2)
	Retried	97 (36.7)
	Other occupations	92 (34.8)
Live alone	Yes	34 (12.9)
	No	230 (87.1)
Surgeries	Yes	237 (89.8)
	No	27 (10.2)
Chemotherapy	Yes	160 (60.6)
	No	104 (39.4)
Radiotherapy	Yes	53 (20.1)
	No	211 (79.9)
Targeted therapy	Yes	33 (12.5)
	No	231 (87.5)
Number of children	≤ 1	100 (37.9)
	2	82 (31.1)
	≥3	82 (31.1)
Course of disease (year)	≤ 1	171 (64.8)
	>1	93 (35.2)
Number of hospitalization	≤ 2	133 (50.4)
	≥3	131 (49.6)
Accompanying chronic diseases	Yes	84 (31.8)
	No	180 (68.2)
Ride time to the healthcare facility	≤ 1 h	113 (42.8)
	>1 h	151 (57.2)
Cost of treatment in last year	< 10 w	128 (48.5)
	≥10 w	136 (51.5)
Total out-of-pocket costs	< 5 w	135 (51.1)
	≥5 w	129 (48.9)
Stage	III	147 (59.8)
	IV	117 (40.2)
Age (years)		56.70 ± 14.70

### The association between FT and demographic variables

The association between FT and demographic variables is shown in Table 2. The finance burden scores were significantly associated with monthly family income (yuan), how medical expenses were paid, current employment status, and whether surgical treatment was received (*P* < 0.05).

**Table 2:** Univariate analysis of FT in patients with middle and advanced CRC ($\bar{x}\pm s$).

**Norm**	**Categorization**	**COST score**	***t/F*-value**	***P*-value**
Sex	Male	15.95 ± 5.78	−1.009	0.314
	Female	16.69 ± 5.92		
Race	Han ethnic group	16.27 ± 5.82	0.020	0.984
	Other ethnic groups	16.25 ± 6.02		
Monthly household income	< 2,000	15.5 ± 5.74	3.382	**0.035** ^*^
	2,000–5,000	16.04 ± 4.77		
	>5,000	17.75 ± 6.81		
Marital status	Married	16.36 ± 5.84	0.590	0.555
	Unmarried/divorced/widowed	15.81 ± 5.88		
Education level	Primary and below	16.16 ± 6.84	2.125	0.097
	Junior high school	17.42 ± 6.21		
	High school/secondary school	15.34 ± 5.05		
	University and above	15.43 ± 4.42		
Payment methods	Self-funded or other	15.56 ± 5.22	2.233	**0.041** ^*^
	Medical insurance	17.92 ± 6.6		
	New Agriculture Cooperative	17.33 ± 6.62		
Employment status	Employed	17.54 ± 6.65		
	Unemployed	14.69 ± 5.36	3.236	**0.023** ^*^
	Retried	14.88 ± 4.79		
	Other occupations	16.13 ± 5.28		
Live alone	Yes	17.12 ± 6.23	0.912	0.363
	No	16.14 ± 5.78		
Surgeries	Yes	16 ± 5.75	2.237	**0.026** ^*^
	No	18.63 ± 6.17		
Chemotherapy	Yes	15.94 ± 5.66	−1.131	0.259
	No	16.77 ± 6.11		
Radiotherapy	Yes	16.2 ± 5.88	−0.340	0.734
	No	16.51 ± 5.72		
Targeted therapy	Yes	16.22 ± 5.47	−0.358	0.721
	No	16.61 ± 8.08		
Number of children (number)	0 or 1	16.44 ± 5.21	1.277	0.280
	2	16.87 ± 6.39		
	≥3	15.45 ± 5.97		
Course of disease (year)	≤ 1	16.37 ± 5.88	0.411	0.681
	>1	16.06 ± 5.8		
Number of hospitalization (frequency)	≤ 2	15.81 ± 5.78	−1.272	0.205
	≥3	16.73 ± 5.89		
Number of concomitant chronic diseases	Yes	15.89 ± 5.84	−1.513	0.131
	No	17.06 ± 5.8		
Ride time to the healthcare facility	≤ 1 h	16 ± 5.59	−0.637	0.524
	>1 h	16.46 ± 6.03		
Cost of treatment in last year	< 10 w	16.35 ± 5.96	0.233	0.816
	≥10 w	16.18 ± 5.75		
Total out-of-pocket costs	< 5 w	16.24 ± 5.88	−0.059	0.953
	≥5 w	16.29 ± 5.82		
Stage	III	19.61 ± 9.3	−0.537	0.325
	IV	19.33 ± 8.1		
Age (years)		17.43 ± 6.81	0.533	0.426

Chi-square tests were conducted on all categorical variables. Statistical significance is indicated by the *P*-value; ^*^Significant (*p* < 0.05).

### Identifying the risk factors of the FT score based on simple linear regression and multiple regression

The relationship between patient population characteristics and cost-effectiveness was further substantiated through the use of linear regression analysis. Tables 2–4 present the development of basic linear regression and multiple regression models. Factors such as high household income, health insurance-based payment methods, employment status, and absence of surgical treatment were identified as protective factors against FT in the univariate analysis. The dependent variable in the multiple linear regression analyses consisted of the FT scores of patients diagnosed with middle and advanced stages of colorectal cancer (CRC). Independent variables identified as statistically significant in univariate and correlation analyses were selected for stepwise regression screening (αin = 0.05, αout = 0.10). Supplementary material displays the initial measurements of medical coping strategies, Anderson symptom evaluation, social support levels, and stigma, along with whether surgical intervention, chemotherapy, radiotherapy, or targeted therapy were utilized, as well as other relevant factors. The findings indicated that five factors, including patients' income, method of medical payment, employment status, severity of symptoms, and overall stigma scale score, were included in the regression model, accounting for 32.4% of the variance (Table 4).

**Table 3:** Analysis of the correlation between the total COST score and the scores of each scale.

**Scale name**	**Score ($\bar{x}\pm s$)**	***r*-value**	***P-*value**
Total COST score	16.27 ± 5.84–	–	–
MCMQTotal score	56.49 ± 4.43	0.035	0.572
Face the dimension	23.72 ± 2.46	0.032	0.605
Avoidance dimension	17.04 ± 2.72	0.041	0.502
Yield dimension	15.74 ± 1.57	−0.023	0.704
Total MDASI score	70.52 ± 32.51	−0.247	**< 0.001** ^*^
Symptom severity dimension	46.53 ± 24.09	−0.295	**< 0.001** ^*^
Symptoms hampering the degree of life	23.98 ± 12.18	0.077	0.212
Total SSCI score	48.37 ± 14.41	−0.375	**< 0.001** ^*^
External Shame	29.69 ± 9.95	−0.021	0.739
External Shame	18.68 ± 5.76	< 0.001	0.998
Total SSRS score	27.46 ± 5.05	−0.131	**0.034** ^*^
Objective support	8.52 ± 2.37	−0.11	0.074
Subjective support	12.27 ± 2.34	−0.115	0.062
Support for utilization	6.68 ± 2.08	−0.063	0.311

*P*-value, level of statistical significance; ^*^Significant (*p* < 0.05).

**Table 4:** Influencing factors of the total COST score as determined by multiple linear regression analysis.

**Variable**	**Ratio β**	**Standard deviation *S.E***	**Standardized factorβ**	***t*-value**	***P*-value**	**95% CI**
(constant)	8.894	1.154	–	7.710	**< 0.001** ^*^	6.623–11.165
Total SSCI score	−0.147	0.026	−0.362	−5.590	**< 0.001** ^*^	−0.095 to −0.198
Symptom severity	−0.175	0.044	−0.721	−3.999	**< 0.001** ^*^	−0.089 to −0.261
Total MDASI score	−0.111	0.033	−0.620	−3.334	**0.001** ^*^	−0.177 to −0.046

Statistical significance is indicated by the *P*-value; ^*^Significant (*p* < 0.05).

## Discussion

This study investigates the presence of FT in individuals with late-stage CRC at a tertiary medical facility in China, while also exploring the factors that may impact it. Our research revealed that 87.1% of individuals diagnosed with colorectal cancer exhibited elevated financial toxicity. Specifically, our findings indicate that individuals without employment and with lower household incomes tend to bear a higher burden of out-of-pocket expenses, with surgical patients being particularly susceptible to experiencing significant financial strain.

We found that the patients with middle and advanced CRC FT has an average FT score of 16.27 ± 5.84, which is lower than the FT score of postsurgical CRC score of patients evaluated by Mo et al. (33). The change of patients' treatment stage may be the reason for this difference. The patients we investigated are in the middle and late treatment stage, with many complications, which leads to relatively high treatment costs, thus making the economic toxicity at a high level. Compared with patients with breast cancer, thyroid cancer, lung cancer and gynecological malignancies (22, 27, 34, 35), patients with intermediate and advanced CRC have higher ft levels, which may be because some patients with intermediate and advanced CRC need to wear ostomy bags for short-term or lifelong after anal diversion surgery. Stoma related accessories are not included in medical insurance, and patients purchase at their own expense, increasing the treatment cost; Secondly, stoma clinics are not popularized in county-level medical institutions, and patients need to go to county-level or municipal or provincial medical institutions to purchase, which increases the transportation costs and economic burden of patients. Most patients with advanced CRC have a long treatment cycle, and some targeted drugs are not included in the medical insurance. The proportion of self payment is high, which further aggravates the economic toxicity of patients. Therefore, medical staff should establish a trust relationship with patients, strengthen communication with patients' medical costs, identify patients with financial difficulties, timely evaluate and manage patients' ft, optimize treatment plans, and control medical cost redundancy; At the same time, the medical and health care departments should also pay attention to the special groups of cancer, incorporate the expensive and commonly used drugs into the medical insurance as soon as possible, improve the reimbursement ratio, and reduce the economic burden of patients with advanced CRC.

Within this research, 15.2% of the participants were without employment, while 36.7% were in retirement, and all individuals in these categories exhibited low COST scores. These findings were in line with those of Pearce et al. (36). Unemployed individuals may face a reduction in monthly household income as a result of diminished financial resources, potentially impeding timely access to medical screening and treatment, thereby delaying medical care and escalating treatment costs and financial strain. Similarly, retirees, despite having a fixed income, the relatively lower income also leads to a decrease in monthly household income, increasing the financial burden. Additionally, research indicated that individuals may experience increased financial burden if their monthly household income is below RMB 2,000 (*P* < 0.05), aligning with the results of Min et al. (37). Many patients do not deposit or have little willingness to deposit. Individuals with low incomes alleviate their financial stress by cutting back on spending in various areas or potentially discontinuing medical treatment.

The allocation of health insurance expenses for individuals who are unemployed or from low-income households is subject to modification. Empirical data suggests that individuals in these demographics may experience adjustments in their contributions toward medical insurance, with some opting to forego payment or reduce their payments as a means of financial conservation (33). Therefore, when the proportion of medical insurance reimbursements decreases and the proportion of self expenses increases, it is more likely to exacerbate FT.

Patients with middle to advanced CRC demonstrate a strong association between symptom severity and financial burden. Symptoms such as cognitive impairment, pain, and tiredness are linked to a decreased ability to reintegrate into society and financial accessibility (38). The results of this study confirm that the severity of symptoms in middle and advanced CRC patients is negatively correlated with FT scores (*r* = −0.295, *P* < 0.001), indicating that the higher the severity of symptoms, the heavier their financial burden, which is consistent with the conclusion by American scholar Chan et al. (38). The aforementioned scenario may be attributed to the fact that individuals with middle to advanced stages of colorectal cancer who present with severe symptoms may necessitate more complex and prolonged treatment modalities, including surgical interventions, chemotherapy, radiation therapy, and targeted pharmacotherapies, resulting in an escalation of healthcare costs. Moreover, the severity of the symptoms may make it impossible for patients to work or carry out daily activities, which may reduce their income and increase their financial burden. Hence, healthcare facilities and insurance companies have the option to lower treatment expenses by negotiating prices or implementing more affordable medication options. Moreover, social welfare systems have the potential to offer financial aid and welfare services to alleviate the financial challenges faced by patients and their families. Additionally, it is imperative for healthcare providers to promptly identify high-risk symptoms, provide psychological support and educational resources, implement appropriate interventions, and continuously monitor treatment progress in order to mitigate cancer-related symptoms, alleviate financial burdens on patients and families, and improve survival rates.

The relationship between stigma and financial burden is influenced by multiple factors (39). Prior research has indicated that individuals in the mid to late stages of colorectal cancer exhibit heightened internal sensitivity, including fears of stoma discovery. These patients frequently conceal or avoid discussing their condition, experience heightened negative emotions during treatment, which may impede their ability to make rational decisions, ultimately delaying treatment initiation and exacerbating disease progression. This in turn contributes to increased treatment complexity and costs (10). Additionally, the stigma surrounding their condition may adversely affect the mental health and overall satisfaction of individuals, hindering their capacity to participate in professional and social activities, consequently resulting in decreased income and financial strain. Furthermore, the stigma may create psychological barriers for patients seeking medical and financial support, causing them to be hesitant in disclosing their circumstances to family, friends, or healthcare professionals, further exacerbating their financial difficulties. The current research indicated a negative relationship between patients' feelings of embarrassment and their financial burden scores (*r* = −0.375, *P* < 0.001). A more severe sense of guilt is linked to lower functional testing scores and higher FT. However, researchers are unable to establish a direct correlation between stigma and financial burden. Therefore, it is imperative to develop appropriate interventions and strategies to help individuals overcome discrimination and mitigate the economic strain on patients with moderate to advanced colorectal cancer.

Currently, although some studies have focused on the issue of financial toxicity in cancer patients, research specifically targeting patients with middle and advanced colorectal cancer remains relatively scarce. Therefore, this study focuses on patients with middle and advanced colorectal cancer, aiming to identify the main factors influencing financial toxicity in these patients. This provides a basis for healthcare institutions and policymakers to develop more targeted interventions, alleviate the financial burden on patients, and improve their quality of life. This research holds significant implications for medical practice and policy formulation.

## Limitations

Given the current conditions and budget constraints, certain limitations must be acknowledged. The sample size of this study is small and limited to a single center located in Guizhou, Southwest China, which exacerbates the issue. The findings may be slightly affected by regional financial disparities and local income levels. There are certain limitations in the statistical methods used, as the interactions between factors and mediation effects were not explored. Future research should include large-scale, multi-center field investigations on cancer, spanning different regions, economic conditions, healthcare policies, and income levels. Collecting data from more patients is necessary to ensure sufficient statistical power to detect interactions and designing longitudinal studies to follow up on patients' financial situations would help better understand the interactions and their dynamic effects. We recognize the value of mediation effect analysis and plan to further explore this direction in future research. We hope to further analyze the role mechanisms of mediating variables through longer follow-up studies and more diverse data collection.

## Conclusions

The findings of this study suggest that individuals diagnosed with middle and advanced stages of colorectal cancer (III-IV CRC) exhibit significantly elevated levels of FT, which appears to be influenced by various factors including the patient's family monthly income, employment status, method of medical payment, severity of symptoms, and the degree of stigma. In view of the highly individualized and subjective characteristics of FT, predicting FT is often challenging. Healthcare professionals should pay attention to the FT levels of patients diagnosed CRC, and implement appropriate intervention strategies. By assessing the financial circumstances of patients, promoting patient involvement in the development and execution of personalized treatment plans, promptly monitoring and addressing patient adverse reactions, and instructing patients on adopting constructive psychological coping strategies to mitigate stigma and lower levels of FT.

## Data availability statement

The original contributions presented in the study are included in the article/Supplementary material, further inquiries can be directed to the corresponding authors.

## Ethics statement

The studies involving humans were approved by Ethics Committee of Guizhou Provincial People's Hospital. The studies were conducted in accordance with the local legislation and institutional requirements. The participants provided their written informed consent to participate in this study.

## Author contributions

XH: Conceptualization, Data curation, Investigation, Project administration, Writing – original draft, Writing – review & editing. JC: Writing – review & editing, Methodology, Investigation, Data curation, Conceptualization. LZ: Methodology, Formal analysis, Conceptualization, Writing – review & editing, Supervision, Project administration. QL: Project administration, Writing – review & editing, Validation, Resources, Funding acquisition, Formal analysis. XZ: Investigation, Resources, Supervision, Validation, Writing – review & editing. JZ: Writing – review & editing. YC: Conceptualization, Data curation, Investigation, Software, Supervision, Writing – review & editing.
