# Supplementary material for: Identifying the factors affecting financial toxicity status in patients with middle and advanced colorectal cancer: a cross-sectional study
Source: Front Public Health. 2024 Jul 16;12:1421314. doi: 10.3389/fpubh.2024.1421314 (PMC11286404; doi:10.3389/fpubh.2024.1421314)
Supplement: Supplementary file 1 [file Data_Sheet_1.PDF]

国家自然科学基金资助项目批准通知

（预算制项目）

李秋萍　　先生/女士：

根据《国家自然科学基金条例》、相关项目管理办法规定和专家评审意见，国家自然科学基金委员会（以下简称自然科学基金委）决定资助您申请的项目。项目批准号：82172844，项目名称：基于患者及配偶共同心理需求的结直肠肿瘤幸存者生存期照护模式研究 ，直接费用：54.00万元，项目起止年月：2022年01月至 2025年 12月，有关项目的评审意见及修改意见附后。

请您尽快登录科学基金网络信息系统（https://isisn.nsfc.gov.cn），**认真阅读《国家自然科学基金资助项目计划书填报说明》并按要求填写《国家自然科学基金资助项目计划书》（以下简称计划书）**。对于有修改意见的项目，请您按修改意见及时调整计划书相关内容；如您对修改意见有异议，须在电子版计划书报送截止日期前向相关科学处提出。

请您将电子版计划书通过科学基金网络信息系统（https://isisn.nsfc.gov.cn）提交，由依托单位审核后提交至自然科学基金委。自然科学基金委审核未通过者，将退回的电子版计划书修改后再行提交；审核通过者，打印纸质版计划书（一式两份，双面打印）并在项目负责人承诺栏签字，由依托单位科研、财务管理等部门审核、签章并在承诺栏加盖依托单位公章，且将申请书纸质签字盖章页订在其中一份计划书之后，一并报送至自然科学基金委项目材料接收工作组。纸质版计划书应当保证与审核通过的电子版计划书内容一致。**自然科学基金委将对申请书纸质签字盖章页进行审核，对存在问题的，允许依托单位进行一次修改或补齐。**

向自然科学基金委提交电子版计划书、报送纸质版计划书并补交申请书纸质签字盖章页截止时间节点如下:

1. **2021年10月22日16点：**提交电子版计划书的截止时间（视为计划书正式提交时间）；

2. **2021年10月29日16点：**提交修改后电子版计划书的截止时间；

3. **2021年11月5日16点：**报送纸质版计划书（其中一份包含申请书纸质签字盖章页）的截止时间。

4. **2021年11月25日16点：**报送修改后的申请书纸质签字盖章页的截止时间。

请按照以上规定及时提交电子版计划书，并报送纸质版计划书和申请书纸质签字盖章页，未说明理由且逾期不报计划书或申请书纸质签字盖章页者，视为自动放弃接受资助；未按要求修改或逾期提交申请书纸质签字盖章页者，将视情况给予暂缓拨付经费等处理。

附件：项目评审意见及修改意见表

国家自然科学基金委员会
2021年10月12日
