# Supplementary material for: Identifying the factors affecting financial toxicity status in patients with middle and advanced colorectal cancer: a cross-sectional study
Source: Front Public Health. 2024 Jul 16;12:1421314. doi: 10.3389/fpubh.2024.1421314 (PMC11286404; doi:10.3389/fpubh.2024.1421314)
Supplement: Supplementary file 2 [file Table_1.DOCX]

**Independent Variable Assignment Method**

| independent variable | Description of the assignment |
| --- | --- |
| Monthly household income | 1= <2000；2= 2000～5000；3= >5000 |
| payment method | 1=Self-funded or otherwise；2=medical insurance；3=New Agricultural Cooperative Society (NACS) |
| Status of work | 1=be employed；2=unemployed；3=retirement ；4=Other professions |
| Surgery or not | 0=No；1=Yes |
| Symptom severity dimension score (points) | continuous variable |
| Total Anderson Symptom Assessment (points) | continuous variable |
| Total score on the morbidity stigma (points) | continuous variable |
| Total FT score (points) | continuous variable |
